# Myelofibrosis Associated with Romiplostim Treatment in a Patient with Immune Thrombocytopenia

**DOI:** 10.1155/2012/318597

**Published:** 2012-04-01

**Authors:** Maria Fernanda Gonzalez, Jonathan King Freeman

**Affiliations:** Department of Pathology, Baystate Medical Center, Tufts University, 759 Chestnut Street, Springfield, MA 01199, USA

## Abstract

Immune thrombocytopenia is characterized by antibody-mediated platelet destruction and insufficient platelet production resulting in isolated thrombocytopenia in the absence of underlying cause. Despite many treatment options, low-to-intermediate rates of remission and high rates of resistance to treatment are seen. Approximately 20% of patients do not attain a hemostatic platelet count after splenectomy or after first- and second-line medical approaches. A new option in these cases is treatment with *romiplostim*. Bone marrow (BM) fibrosis has been reported in clinical trials with romiplostim. We report a case with marked reticulin fibrosis of the BM, worsening of cytopenias and dyserythropoiesis, and atypical megakaryocytes, which did not reverse following cessation of the drug. Although this could represent idiopathic myelofibrosis, unrelated to therapy, the pretreatment biopsy demonstrating no fibrosis combined with the concordant timing of the drug and fibrosis suggests the treatment with romiplostim may be causative.

## 1. Introduction

Immune thrombocytopenia is characterized by antibody-mediated platelet destruction and insufficient platelet production [1, 2] resulting in isolated thrombocytopenia in the absence of underlying cause [3]. Usually, ITP is a diagnosis of exclusion, after immune and nonimmune causes of thrombocytopenia, including disseminated intravascular coagulation, connective tissue diseases, lymphoproliferative disorders, infection, mechanical destruction, drugs, thrombotic thrombocytopenia purpura, and certain alloantibody-mediated thrombocytopenias have been ruled out [4].

Treatment is rarely necessary in patients with platelet counts above 50 k/mm^3^, unless accompanied by other risks for bleeding, including platelet dysfunction or another hemostatic defect, trauma, surgery, mandated anticoagulation therapy, or in persons whose profession or lifestyle predisposes them to trauma [2].

First-line treatment for ITP includes corticosteroids, intravenous anti-D, and intravenous immunoglobulins. Second-line treatment includes azathioprine, cyclosporin A, cyclophosphamide, danazol, dapsone, mycophenolate mofetil, rituximab, and splenectomy [2]. Despite all the treatment options, low-to-intermediate rates of remission and high rates of resistance to treatment are seen [3, 5]. 10% to 20% of splenectomy responders eventually relapse [2, 5]. Approximately 20% of patients do not attain a hemostatic platelet count after splenectomy or after first- and second-line medical approaches [2]. Treatment options for adult patients failing to these initial treatments include combination chemotherapy, hematopoietic stem cell transplantation, and thrombopoietin-receptor agonists as* romiplostim and eltrombopag* [2]. Clinical trials have proved high efficacy to these new agents, (86-87% response), and possible long-term maintenance of remission using interrupted administration of low doses [5].

Romiplostim is an FDA-approved treatment of adults with chronic ITP [6]. Romiplostim is a fusion protein produced by DNA technology that contains two copies of the constant fraction of immunoglobulin G_1_, each of which is covalently linked to a peptide chain containing two thrombopoietin receptor-binding peptides [7]. This agent is a thrombopoiesis-stimulating protein that mimics endogenous thrombopoietin (TPO) by binding to the human TPO receptor, leading to activation of downstream signaling via the JAK-STAT pathway that results in increased platelet production [1, 2, 7, 8]. Romiplostim does not interfere with antibody production or binding to platelets [8]. Romiplostim has been shown to improve platelet counts during both short- and long-term use in adult patients with chronic ITP [1]. The most frequently reported adverse events have been arthralgias (26%), fatigue (13%), and nausea (7%). Increased thromboembolic risk has been associated to the use of romiplostim [9]. Bone marrow fibrosis has been reported in clinical trials with romiplostim [1]. 10 out of 271 patients were reported to have reticulin deposition [1], but the fibrosis was reversible and dose dependent [1]. There have been no reported cases of irreversible myelofibrosis in which thrombopoietic agents have been clearly implicated in causation [10].

## 2. Case History

A 76-year-old male was diagnosed with immune thrombocytopenic purpura (ITP) following a six-month history of fatigue, increasing bruising on his upper and lower extremities and gum bleeding. His past medical history included type 2 diabetes mellitus, hypertension, atrial fibrillation, coronary artery disease, and ischemic cardiomyopathy. He denied fevers, night sweats, or weight loss. His physical examination did not show splenomegaly. His medications included lisinopril, spironolactone, sotalol, Coreg, Lipitor, aspirin 81 mg per day, insulin, and warfarin. A complete blood count showed hemoglobin of 10.4 gm/dL, mean corpuscular volume of 96.2 femtoliters, red blood cell count of 3.40 m/mm^3^, and platelets of 37 k/mm^3^.

**Figure 1:**
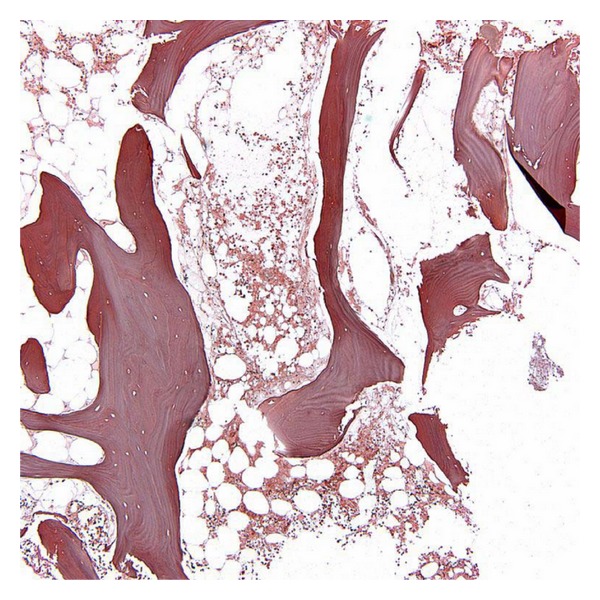
Bone marrow biopsy before treatment with romiplostim showing the absence of reticulin fibrosis (Silver stain, original magnification × 400).

The patient was followed with periodic blood counts for five months without treatment, but demonstrated progressive thrombocytopenia for which he was referred to a hematologist. A bone marrow examination was performed showing mildly hypercellular marrow for age (50–60% cellularity), without fibrosis (Figures 1, 3, 5 and 7). Cytogenetic results demonstrated a normal karyotype. The patient was diagnosed with ITP and superimposed anemia. Coumadin was discontinued, and the patient started treatment with rituximab. For the following eight weeks, he showed no response, with platelet count remaining in the mid 30 k/mm^3^ range.

**Figure 2:**
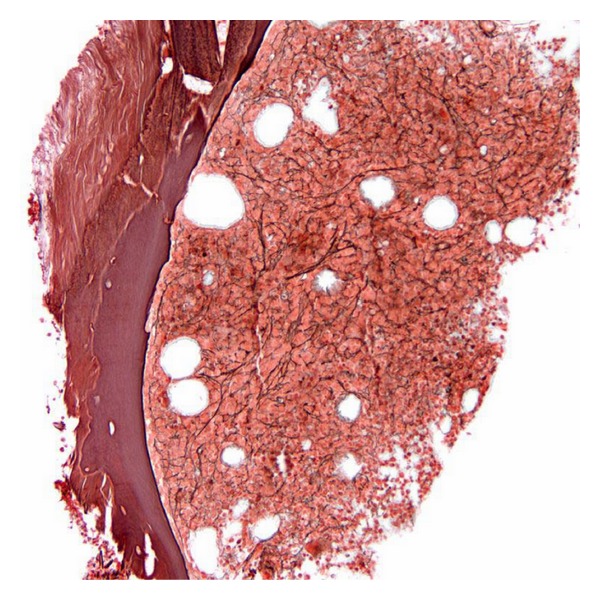
Bone marrow biopsy after treatment with romiplostim demonstrating marked diffuse reticulin fibrosis (Note coarse reticulin fiber network) (Silver stain, original magnification × 400).

**Figure 3:**
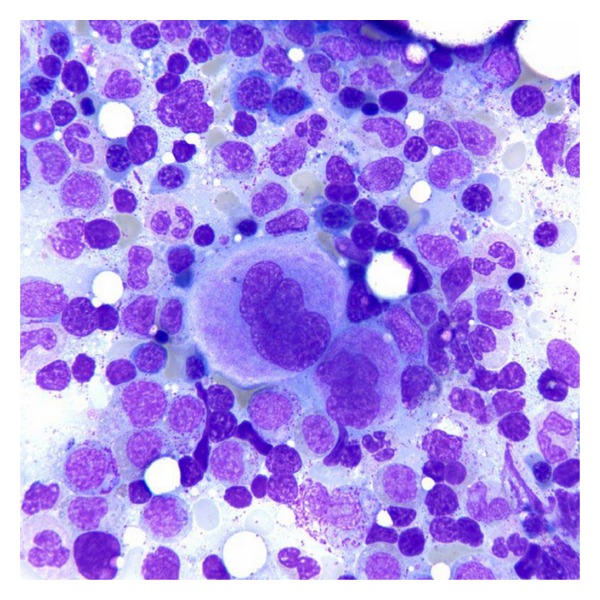
Bone marrow aspirate with normal megakaryocytes (Wright-Giemsa, original magnification × 100).

**Figure 4:**
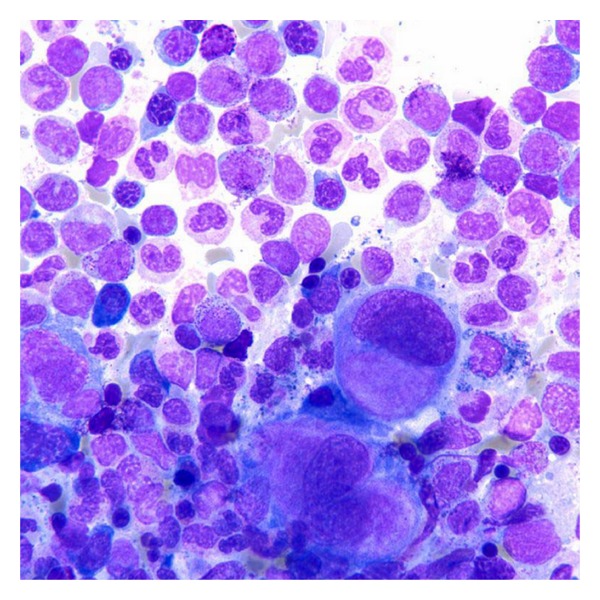
Bone marrow aspirate after treatment with romiplostim with hypolobated megakaryocytes (Wright-Giemsa, original magnification × 100).

**Figure 5:**
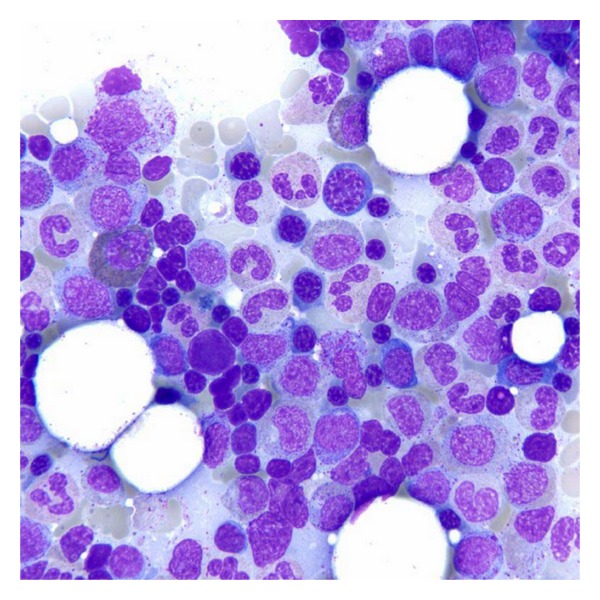
Bone marrow aspirate with normal erythropoiesis (Wright-Giemsa, original magnification × 100).

**Figure 6:**
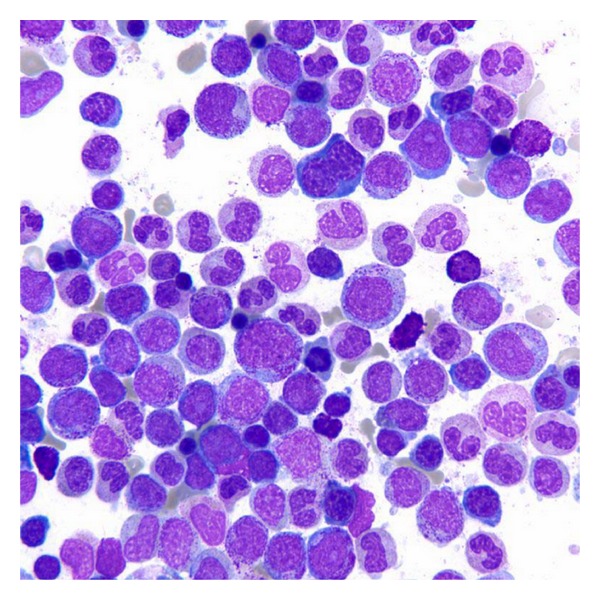
Bone marrow aspirate after treatment with romiplostim with erythrodysplasia (Note binucleation, irregular nuclear contours, and dyssynchronic maturation of the erythroid line) (Wright-Giemsa, original magnification × 1000).

**Figure 7:**
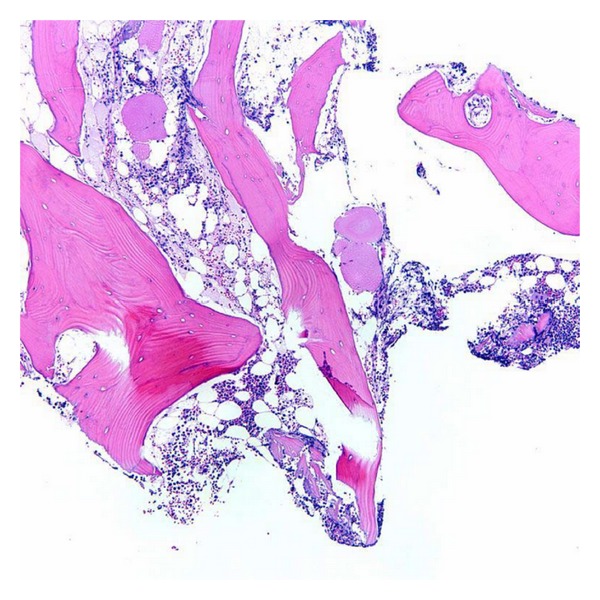
Mildly hypercellular bone marrow for age (50–60% cellularity) (hematoxylin-eosin, original magnification × 100).

**Figure 8:**
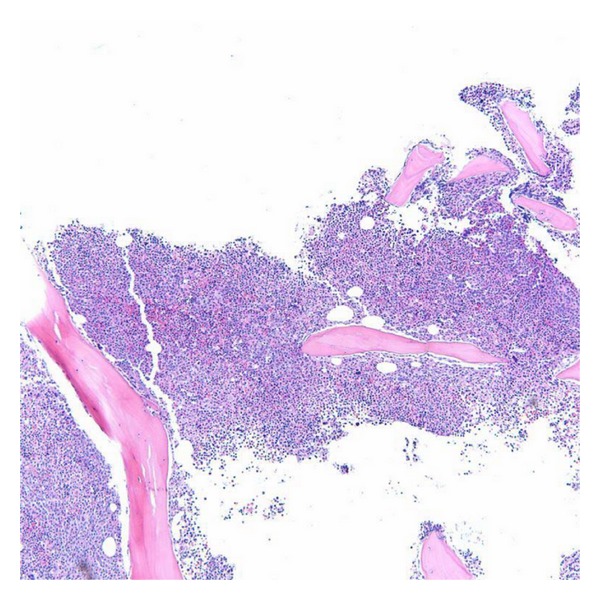
Marked hypercellular bone marrow for age (90% cellularity), with slight dyserythropoiesis (hematoxylin-eosin, original magnification × 40).

Due to his cardiac condition, the patient was not a candidate for splenectomy and instead started treatment with low dose of romiplostim (100 mcg subcutaneous injections every seven days during 5 months). Initially, the patient showed response to the treatment, with platelet counts increasing to between 60–100 k/mm^3^. However, over the course of the following year, his platelet counts progressively decreased, despite increasing doses of romiplostim to 300 and 400 mcg/week. During this time, the patient also developed worsening anemia, to hemoglobin 8.5 gm/dL, requiring transfusion of red blood cells on two occasions in the last 3 months. Along with the worsening cytopenias, the patient developed increasing fatigue and shortness of breath but remained without splenomegaly. A repeat bone marrow biopsy was performed showing markedly increased cellularity (90% cellularity), slight dyserythropoiesis, occasional atypical megakaryocytes, and now with a marked diffuse reticulin fibrosis (Figures 2, 4, 6 and 8). Repeat karyotype remained normal. Fluorescence in situ hybridization failed to demonstrate deletion of chromosome 5 or 7.

## 3. Comment

The major differential diagnosis of marrow fibrosis in this setting includes primary myelofibrosis, fibrosis in ITP, myelodysplastic syndrome (MDS) with myelofibrosis, infectious/granulomatous reactions, acute panmyelosis with myelofibrosis, and autoimmune diseases [11]. To accurately distinguish among these possibilities, correlation of the clinical history, laboratory findings, and the histology of the bone marrow is critical.

Our patient showed a marked reticulin fibrosis of the bone marrow, worsening of his cytopenias, dyserythropoiesis, and atypical megakaryocytes. After discontinuing romiplostim, his cytopenias have not corrected. The patient has required transfusion of red blood cells despite cessation of the drug. Although this could represent idiopathic myelofibrosis (primary MDS), unrelated to therapy, the pretreament biopsy demonstrating no fibrosis combined with the concordant timing of the drug and fibrosis suggests the treatment with romiplostim may have been causative.

## References

[1] Kuter DJ, Mufti GJ, Bain BJ, Hasserjian RP, Davis W, Rutstein M (2009). Evaluation of bone marrow reticulin formation in chronic immune thrombocytopenia patients treated with romiplostim. *Blood*.

[2] Provan D, Stasi R, Newland AC (2010). International consensus report on the investigation and management of primary immune thrombocytopenia. *Blood*.

[3] Leung T, Lokan J, Turner P, Smith C (2011). Reversible bone marrow reticulin fibrosis as a side effect of romiplostim therapy for the treatment of chronic refractory immune thrombocytopenia. *Pathology*.

[4] Rodgers G, Kjeldsberg C, Perkins S (2009). Thrombocytopenia. *Practical Diagnosis of Hematologic Disorders*.

[5] Kovaleva LG, Pustovaia EI, Safonova TI, Riadnenko AA, Kolosova EN (2011). Clinical statistics and effectiveness of different treatments of idiopathic thrombocytopenic purpura. *Terapevticheskii Arkhiv*.

[6] Khellaf M, Michel M, Quittet P (2011). Romiplostim safety and efficacy for immune thrombocytopenia in clinical practice: 2-year results of 72 adults in a romiplostim compassionate-use program. *Blood*.

[7] Wang Y-MC, Sloey B, Wong T, Khandelwal P, Melara R, Sun Y-N (2011). Investigation of the pharmacokinetics of romiplostim in rodents with a focus on the clearance mechanism. *Pharmaceutical Research*.

[8] Despotovic JM, Ware RE (2011). Thrombopoiesis: new ITP paradigm?. *Blood*.

[9] Rayoo R, Sharma N, van Gaal WJ (2011). A case of acute stent thrombosis during treatment with the thrombopoietin receptor agonist peptide-romiplostim. *Annals of Hematology*.

[10] McKrae K (2011). Immune thrombocytopenia: no longer ‘idiopathic’. *Cleveland Clinic Journal of Medicine*.

[11] Anastasi J, Vardiman J, Kjeldsberg C, Perkins S (2009). Primary myelofibrosis. *Practical Diagnosis of Hematologic Disorders*.

